# Magnitude of Induced Abortion and Associated Factors among Female Students of Hawassa University, Southern Region, Ethiopia, 2019

**DOI:** 10.1155/2020/2856502

**Published:** 2020-09-22

**Authors:** Addisu Tadesse Sahile, Mieraf Shiferaw Beyene

**Affiliations:** ^1^Department of Public Health, Unity University, Addis Ababa, Ethiopia; ^2^Department of Public Health, Universal Medical College, Addis Ababa, Ethiopia

## Abstract

**Objectives:**

This study was aimed at assessing the magnitude of induced abortion and associated factors among students in Hawassa University, southern region, Ethiopia, 2019.

**Methods:**

An institutional-based cross-sectional study was conducted among a total of 422 students selected on the bases of a probability simple random sampling method. A pretested structured questionnaire was used to collect data. Analysis was made with SPSS 20. Descriptive summary and inferential statistics (binary logistic regression) were used with a 95% CI and *P* value of less than 5% as a level of significance. Findings were presented in tables, figure, and texts. Confidentiality of information was also secured.

**Results:**

The prevalence of induced abortion in the study setting was 68.7% (95% CI: 64.15%-73.2%). Participants who used emergency contraceptives had 12 times higher odds of undergoing abortion than those who did not use emergency contraceptives at AOR: 11.95, 95% CI: 5.615-25.326, *P* < 001.

**Conclusions:**

A higher prevalence of induced abortion was observed in the study setting. Contraceptive use was the predictor of induced abortion identified. Concerned bodies were recommended to work on the identified determinant of induced abortion in the study setting.

## 1. Introduction

Abortion has been an old experience carried out so far by human beings. It has been practiced throughout the world illegally. Globally, it was estimated that around 30 million induced abortions were performed annually [1]. Globally, from 210 million pregnancies that occur annually, about 22% end up in induced abortion [2].

In sub-Saharan Africa (SSA), many women use abortion as a means of family planning methods. With restrictive abortion laws and limited contraceptive access, unsafe abortion accounted for 13% of maternal deaths in SSA [3]. About 9% of maternal deaths in sub-Saharan Africa are attributed to complications of unsafe abortion [4].

An estimated 620,300 induced abortions were performed in Ethiopia annually. The annual abortion rate was 28 per 1,000 women aged 15-49, with the highest in urban areas [5]. Induced abortion is one of the mechanisms to deal with unwanted pregnancy. The university students in Ethiopia dealt with unwanted pregnancy by undertaking induced abortion to terminate pregnancy secretly to avoid stigma following premarital pregnancy [6].

Ethiopia is one of the countries that allowed women to obtain safe and legal abortion under some conditions; these conditions included the following: if the pregnancy was from rape, if there is physical or mental disability, if it would put women on physical health or life risk, or if the woman is younger than 18 and unprepared to give birth [7].

Though most studies in Ethiopia were health facility-based on patients seeking health service, reproductive health service, particularly abortion-related issues, was not well emphasized. So, the current study assessed the magnitude of induced abortion and associated factors among students at Hawassa University during the year 2019.

## 2. Subjects and Methods

### 2.1. Study Setting and Period

The study was conducted in Hawassa University, located in Southern Nations, Nationalities, and People's Region (SNNPR). It is located 278 kilometers south of Addis Ababa, the capital of Ethiopia. The university is one of the governmental universities located in the southern region of Ethiopia. The university has 7 colleges, with a total of 155,965 populations of which 19,500 were female population. The study was conducted from February to May 2019. An institutional-based cross-sectional study was done. The female students in Hawassa University during the year of 2018/2019 were the source population whereas female students of Hawassa University who were available from February to May 2019 were the study population. Participants were selected on the basis of a simple random sampling technique, where the sampling frame was once determined from registries of the university. Though there were conducted studies, the researcher took 50%, as an estimator of prevalence of induced abortion, to have the maximum sample size. A single population proportion formula was utilized to compute the sample size with its respective standard deviation and margin of error. Accordingly, with the consideration of a 10% nonresponse rate, the final sample was found at 422. A structured pretested self-administered questionnaire was employed among the participants in Amharic (original language) after checking for consistency with English by linguistic professionals. The tool was first developed by the researchers after rigorous reviewing of literatures on the topic, and then, inputs of senior researchers were incorporated into the developed tool. For the coherence, clarity, and conciseness of the questionnaire, native speakers reviewed the tool. The outcome variable was induced abortion measured as follows: abortion is termination of pregnancy before 28 weeks of pregnancy [8]. For the purpose of the current study, induced abortion is “students who terminated their pregnancy intentionally either on their own or by another person” [9].

### 2.2. Statistical Analysis

Data was entered and further analyzed with SPSS version 20. Descriptive statistics were employed as a summary measure. Associations between covariates and dependent variables were investigated with binary logistic regression with a 95% confidence interval (95% CI) and *P* value less than 5% as the level of significance. Bivariate logistic regression was done first; then, to take control of effects of confounding variables, multivariate logistic regression was also done.

## 3. Results

### 3.1. Sociodemographic Characteristics of the Respondents

Four hundred twenty-two participants participated in this study with a 100% response rate. Regarding years of education, 17%, 41.2%, 30.6%, 8.8%, and 5.5% of the students were 1^st^ year, 2^nd^ year, 3^rd^ year, 4^th^ year, and 5^th^ year students, respectively. Concerning the departments, 32% were engineering, 15.6% were health, and 33.4% were social science, and the remaining 11.1% were agriculture students. More than half (61.6%) of students lived in the campus dormitory; 37.7% of students lived outside the campus. Less than half (42.2%) of students got income from their parents, 29.9% of the students got an income from relatives, and 27.7% of students got income from other sources (Table 1).

### 3.2. Behavioral Characteristics of Participants

Eighty-two percent of students had history of sexual intercourse. The reasons behind their start of sexual intercourse were the following: 40.3% influence of economic problems, 46.4% peer pressure, 44.1% alcohol consumption, 38.9% personal desire, and 34.8 influence of khat or drugs (Table 2).

### 3.3. Participant's Knowledge about Abortion

As to respondent view about whether participants had heard about emergency contraceptives, majority 406 (96%) had information about it, with most 326 (77%) of the respondents having used the drugs. More than half 265 (62%) of respondents reported that they knew where abortion is legally conducted. Most (77%) of the respondents reported that they used emergency contraceptives following sexual intercourse. Regarding the experience of abortion by the study participants, most (69%) of the participants reported that they had undertaken induced abortion, of which more than half (54%) of the participants used medication for termination of the pregnancy. More than half (53%) of the participants reported that holding off sexual intercourse was their choice when their partner was unwilling to use a condom (Table 3).

### 3.4. Magnitude and Associated Factors of Induced Abortion

**Figure 1:**
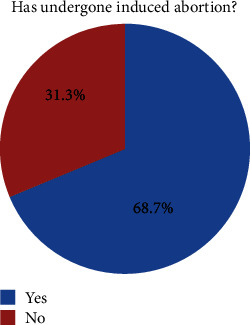
Magnitude of induced abortion of female students at Hawassa University, SNNPR, Ethiopia, April 2019.

The magnitude of induced abortion in the study setting was 68.7% (95% CI: 64.15%-73.2%), whereas only less than one-third (31.3%) did not undergo induced abortion (Figure 1). The age of participants, year of education, alcohol use, and use of emergency contraceptives have independently shown a statistical association with the occurrence of induced abortion at *P* value less than 5%. But in the multivariate logistic regression, only emergency contraceptive use was statistically associated with the occurrence of induced abortion. Participants who used emergency contraceptives had 12 times higher odds of taking induced abortion than those who did not use emergency contraceptives (AOR: 11.95, 95% CI: 5.615-25.326, *P* < 0.001) (Table 4).

## 4. Discussion

The current study found a higher prevalence of induced abortion among university students observed at 68.7%, which was much higher than the findings of the health facility-based study in Guraghe zone of Ethiopia, which revealed the magnitude of induced abortion at 12.3% [9]. This variation might be due to variation in study settings.

The other study in Ethiopian university students revealed that the rate of induced abortion was found at 65 per 1,000 women. Students with history of alcohol use and first year students had higher risk of abortion than their counterparts. About 23.7% of students reported experience of sexual intercourse, and less than half (44%) of respondents reported ever hearing of emergency contraception, of which 36% of those with sexual experience ever used a condom [10], which was lower than the findings of the current study.

The prevalence of induced abortion among precollege students in Ethiopia was observed at 13.6% [11], which was lower than the findings of the current study. The finding from this study was much higher than the findings in China 8.13% [12], Cameroon 21% [13], southern Ethiopia 43.4% [14], Nigeria 51% [15], and Northwestern Ethiopia 4.8% [16]. This variation might be attributed to differences in the time of investigation of sample size, whereas the current finding was almost consistent with the study in Ghana 64% [17].

Having more than four pregnancies (AOR = 4.28, 95%CI = 1.24‐14.71) and age 30-34 years (AOR = 0.15, 95%CI = 0.04‐0.55) were found to be statistically associated with induced abortion [9]. But in the current study, contraceptive use was the only predictor of induced abortion.

In the other study, being in rural areas (OR = 1.21, 95% CI: 1.04–1.39), ages 18-25 (OR = 0.84, 95% CI: 0.72–0.99) and 30 or older (OR = 1.63, 95% CI: 1.42–1.86), and single individuals (OR = 1.72, 95% CI: 1.05–2.83) were more likely to experience induced abortion [12], where none of the variables showed any statistical association in the current study.

Evidences suggested that history of abortion and use of contraceptive methods were statistically interrelated with each other. Women who had history of abortion were more likely to be those using any methods of contraceptives. A study in Luanda, Angola, supported that history of induced abortion was associated with the use of contraceptive methods. Those women who had a history of induced abortion were 1.23 times more likely to use a modern contraceptive method as compared to those who never had abortion (RR: 1.23, 95% CI: 1.10-1.36) [18].

Desire to stay in school (28%), fear of parents (24%), and shame of being pregnant (26%) were the major depicted determinants of induced abortion. Most of the participants were not aware of where abortion is allowed, and some of them had undertaken illegal abortion [13]. The other study in Cameroon depicted that the prevalence of induced abortion was found at 25% [19], of which the finding was lower than the finding of the current study.

The prevalence of induced abortion in this study was higher than findings from the following: 33.6% Addis Ababa, Ethiopia [20], 5% in Pakistan [21], and 17% in Iran [22].

A study at Felege Hiwot Hospital, Ethiopia, revealed that being nonmarried and student, age less than 24 years, and having previous history of induced abortion and low monthly income were independent predictors of induced abortion [23], whilst in this study, only emergency contraceptive use was statistically associated with the occurrence of induced abortion.

In the current study, participants who used emergency contraceptives had 12 times higher odds of undergoing abortion than those who did not use emergency contraceptives. Surprisingly, a higher magnitude of induced abortion was observed from the current study than most other previous studies.

## 5. Conclusions

A higher prevalence of induced abortion was observed in the study setting. Contraceptive use was the predictor of induced abortion. Participants who used emergency contraceptives had 12 times higher odds of undergoing induced abortion than those who did not use emergency contraceptives. Interventions focused on identified determinants could be recommended.

## Figures and Tables

**Table 1:** Sociodemographic characteristics of female students in Hawassa University, SNNPR, Ethiopia, April 2019.

Characteristics	Categories	Number	%
Age in years	18-20	25	5.9
	21-23	344	81.5
	24-26	53	12.6

Year of education	1^st^ year	74	17.5
	2^nd^ year	174	41.2
	3^rd^ year	129	30.6
	4^th^ year	37	8.8
	5^th^ year	8	1.9

Respondent department	Engineering	135	32.0
	Health	66	15.6
	Social science	141	33.4
	Agriculture	47	11.1
	Other	33	7.8

Respondent places they live	In the campus dormitory	260	61.6
	Outside the campus	159	37.7
	With my parents	3	7

Respondent source of income	Parents	179	42.4
	Relatives	126	29.9
	Others^∗^	117	27.7

^∗^Friends, boyfriends, and sugar daddy.

**Table 2:** Behavioral characteristics of female students at Hawassa University, SNNPR, Ethiopia, April 2019.

Variables	Categories	Frequency	%
Had history of sexual intercourse	Yes	347	82.2
	No	75	17.8
Peer pressure was the reason for intention to have sexual intercourse	Yes	196	46.4
	No	226	53.6
Personal desire was the reason for intention to have sexual intercourse	Yes	164	38.9
	No	258	61.1
Influence of alcohol was the reason for initiation of sexual intercourse	Yes	186	44.1
	No	236	55.9
Influence of khat or drug was the reason for initiation of sexual intercourse	Yes	147	34.8
	No	275	65.2
Economic problem was the reason for initiation of sexual intercourse	Yes	170	40.3
	No	252	59.7
Other factors were the reason for initiation of sexual intercourse	Yes	169	40.0
	No	253	60.0

**Table 3:** Participants' knowledge of a place and complication of abortion at Hawassa University, SNNPR, Ethiopia, April 2019.

Characteristics		Number	%
Have you heard about emergency contraceptive drug?	Yes	406	96.2
	No	9	2.1
	Not sure	7	1.7

Did you use emergency contraceptive following sex?	Yes	326	77.3
	No	96	22.7

Have you had abortion?	Yes	290	68.7
	No	132	31.3

Reasons for undertaking abortion	Because it affects my education	98	33.7
	Because I cannot raise a child	96	33.1
	To protect social stigma	65	22.4
	Because I got pregnant due to sexual assault	22	7.6
	Others	11	3.2

Did you know where abortion is performed legally?	Yes	265	62.8
	No	156	37.0
	Am not sure	1	0.2

Respondent place of performing abortion	Community pharmacy	76	26.4
	Health center	104	35.8
	Private clinic	109	37.5
	Traditional	1	0.3

How long since undertaking of abortion?	1 month	72	24.8
	2 months	114	39.3
	3 months	90	31.0
	Do not know	14	4.9

Did you face infection during abortion?	Yes	87	30.0
	No	203	70.0

Did you face bleeding during abortion?	Yes	15	5.1
	No	275	94.9

Type of procedure	Medication	156	53.8
	Instrument	134	46.2

If your partner does not volunteer to use a condom, what would you do?	Stop sexual intercourse	222	52.6
	Try to convince them to use condom	177	41.9
	Practice sexual intercourse without using condom	23	5.5

**Table 4:** Factors associated with induced abortion among Hawassa University students, SNNPR, Ethiopia, April 2019.

Characteristics		Abortion		COR (95% CI)	*P* value	AOR (95% CI)	*P* value
		Yes	No				
Age (grouped)	18-20	11	14	1	0.015	1	0.369
	21-23	238	106	0.350 (0.154-0.796)	0.012	2.546 (0.682-9.505)	0.164
	24-26	41	12	0.230 (0.083-0.637)	0.005	2.717 (0.588-12.558)	0.201

Years of education	1^st^ year	34	40	1	0.000	1	0.023
	2^nd^ year	122	52	0.362 (0.207-0.635)	0.000	7.862 (0.437-141.384)	0.162
	3^rd^ year	96	33	0.292 (0.160-0.535)	0.000	4.276 (0.249-73.451)	0.317
	4^th^ year	31	6	0.165 (0.061-0.441)	0.000	3.753 (0.219-64.422)	0.362
	5^th^ year	7	1	0.121 (0.014-1.037)	0.054	0.816 (0.040-16.617)	0.895

Monthly income in birr	300-500	48	29	1	0.561	1	0.270
	501-700	117	52	0.736 (0.418-1.294)	0.287	0.571 (0.274-1.190)	0.135
	701-900	106	42	0.656 (0.366-1.175)	0.156	0.473 (0.216-1.032)	0.060
	≥901	19	9	0.784 (0.313-1.962)	0.603	0.837 (0.269-2.607)	0.759

Alcohol use	Yes	152	34	3.175 (2.018-4.994)	0.000	0.730 (417-1.278)	0.271
	No	138	98				

Use of emergency contraceptives	Yes	272	54	21.827 (12.101-39.370)	0.000	11.95 (5.615-25.326)	0.000
	No	18	78				

Have reproductive health education	Yes	79	29	1.330 (0.818-2.163)	0.251	0.846 (0.453-1.580)	0.600
	No	211	103				

## Data Availability

All the required data has been included within the manuscript.
